# Morselized Bone Graft: A Tool for Nasal Dorsum Contouring and Refinement

**DOI:** 10.1093/asjof/ojaf018.007

**Published:** 2025-05-13

**Authors:** Shaishav Datta, Steven Hanna, Bugra Tugertimur, Alexia Lucas, Alannah Phelan, Matthew Morris, Paige Goote, David Mattos, Richard Reish

**Affiliations:** University of Toronto, Toronto, ON, Canada; Private Practice, Toronto, ON, Canada; New York Plastic Surgical Group, New York, NY; New York Plastic Surgical Group, New York, NY; Manhattan Eye, Ear and Throat Hospital, New York, NY; New York Plastic Surgical Group, New York, NY; Manhattan Eye, Ear and Throat Hospital, New York, NY; New York Plastic Surgical Group, New York, NY; Manhattan Eye, Ear and Throat Hospital, New York, NY; New York Plastic Surgical Group, New York, NY; Manhattan Eye, Ear and Throat Hospital, New York, NY

## Abstract

**Goals/Purpose:**

The nasal dorsum is a cornerstone of rhinoplasty aesthetics, playing a vital role in achieving facial harmony and balance. Achieving a smooth, refined nasal profile remains a significant challenge, particularly in thin-skinned patients who are more susceptible to contour irregularities. Many techniques are used to address this problem, including diced cartilage, fascia, acellular dermal matrices, and silicone implants. This study aims to evaluate the effectiveness of using morselized bone grafts (MBG)–specifically, unused bone rasp material that is typically discarded–as a technique for contouring and refining the nasal dorsum after dorsal reduction.

**Methods/Technique:**

The senior surgeon exclusively utilizes an open approach to rhinoplasty, and all cases are performed under general anesthesia. After performing a dorsal hump reduction with a bone rasp, the MBG is stored on the back table for later use in the same case. After addressing other components of the rhinoplasty procedure, attention is turned back to the nasal dorsum, wherein any contour irregularities are filled with the MBG paste.

A retrospective chart review of rhinoplasty cases in the senior author's practice was conducted between January 2021 and June 2022. The inclusion criteria were cosmetic or functional rhinoplasty cases in which autologous MBG was used for dorsum refinement and contouring with a minimum of 12 months of follow-up. 953 patients met the inclusion criteria and were included in the study. Outcomes of interest included the rate of postoperative infection, defined as patients with signs of infection requiring antibiotics after completing their standard course of prophylactic antibiotics, and the rate of operative revisions.

**Results/Complications:**

The mean age of our study group was 31.6 years old, with 869 female patients. 640 cases were primary rhinoplasties. The mean follow-up period was 23.5 months, with a minimum of 12 months of follow up for each patient. The rate of postoperative infection in our case series was 2.7%, with 26 patients requiring postoperative antibiotics. 17 (1.8%) patients required operative revision, among whom 4 (23.5%) patients were revision cases. There were no patients who sought revision rhinoplasty for concerns related to dorsal irregularities or contour defects.

**Conclusion:**

MBG use for nasal dorsum aesthetics is a safe, convenient, and effective technique in camouflaging and concealing nasal dorsum irregularities in both primary and revision rhinoplasty. Additionally, as there is no additional equipment required and minimal operative time added when performing this technique, MBG use is an efficient alternative to other techniques for addressing dorsal aesthetics with no additional donor-site morbidity when paired with boney dorsal reduction. Future steps will involve performing a five-year follow-up on this cohort of patients to assess for long-term dorsal aesthetics following MBG use.

